# What is the correlation between the defective and splitting posterior segmental bronchus and recurrent artery crossing intersegmental planes in the right upper lobe?

**DOI:** 10.3389/fsurg.2023.1113783

**Published:** 2023-02-13

**Authors:** Zhikai Li, Yuhong Kong, Wenbo Wu, Shuangqing Chen, Xiaopeng Zhang

**Affiliations:** ^1^ Graduate School, Hebei Medical University, Shijiazhuang, China; ^2^ Department of Thoracic Surgery, Hebei General Hospital, Shijiazhuang, China; ^3^ Graduate School, Hebei North University, Zhangjiakou, China

**Keywords:** right upper lobe (RUL), anatomical variation, recurrent artery crossing intersegmental planes, non-small cell lung carcinoma (NSCLC), segmentectomy, video-assisted thoracoscopic surgery

## Abstract

**Background:**

With the prevalence of three-dimensional computed tomography bronchography and angiography (3D-CTBA) and the development of anatomical segmentectomy, studies have confirmed the increased incidence of anomalous veins in patients with tracheobronchial abnormalities. Nevertheless, the characteristic anatomical correlation between bronchus and artery variation remains unknown. Thus, we conducted a retrospective study to investigate recurrent artery crossing intersegmental planes and their associated pulmonary anatomical features by analyzing the incidence and types of the right upper lobe (RUL) bronchus and the artery composition of the posterior segment.

**Materials and Methods:**

A total of 600 patients with ground-glass opacity who had undergone 3D-CTBA preoperatively at Hebei General Hospital between September 2020 and September 2022 were included. We reviewed the anatomical variations of the RUL bronchus and artery in these patients using 3D-CTBA images.

**Results:**

Among all 600 cases, the defective and splitting B2 contained four types of the RUL bronchial structure: B1 + BX2a, B2b, B3 (11/600, 1.8%); B1, B2a, BX2b + B3 (3/600, 0.5%); B1 + BX2a, B3 + BX2b (18/600, 3%); B1, B2a, B2b, B3 type (29/600, 4.8%). The incidence of recurrent artery crossing intersegmental planes was 12.7% (70/600). The incidence of recurrent artery crossing intersegmental planes with and without the defective and splitting B2 was 26.2% (16/61) and 10.0% (54/539), respectively (*p* < 0.005).

**Conclusions:**

In patients with defective and splitting B2, the incidence of recurrent artery crossing intersegmental planes was increased. Our study provides certain references that surgeons can use to plan and perform RUL segmentectomy.

## Introduction

An increasing number of ground-glass opacities (GGOs) are being detected as high-resolution computed tomography (HRCT) technology advances. Several studies have indicated that segmentectomy is non-inferior to lobectomy when treating lesions with a diameter of less than 2 cm and with a consolidation tumor ratio of less than 25% in terms of 5-year overall survival (1–5); therefore, video-assisted thoracoscopic surgery (VATS) segmentectomy will be an option for treating early-stage non-small cell lung carcinoma (NSCLC) in the upcoming years. Anatomical variations of the peripheral vessels and bronchi are often encountered during segmentectomy, presenting great difficulties and challenges compared to lobectomy. Thus, a comprehensive understanding of pulmonary bronchovascular patterns is particularly essential to the implementation of safe and accurate segmentectomy.

In recent years, three-dimensional (3D) preoperative reconstruction from two-dimensional (2D) images has become more popular due to the advancement of technology, thus, allowing thoracic surgeons to visualize pulmonary anatomical structure before the procedure. Several reports have focused on the use of 3D computed tomography bronchography and angiography (3D-CTBA) to describe variations in the right upper lobe (RUL) bronchovascular patterns and confirm the increased incidence of the right top pulmonary vein in patients with tracheobronchial abnormalities (6–10). However, no reports have described the correlation between a pulmonary bronchus and artery variations. In our clinical practice, the anatomical variation of the RUL bronchus does not conform with the previous findings (6–8). Therefore, we conducted this study. This study aimed to analyze RUL pulmonary bronchus patterns by using data derived from 3D-CTBA and compare the results of the Chinese population with those of similar studies that have been previously conducted abroad. Furthermore, recurrent artery crossing intersegmental planes, which have not been reported in previous studies, were found in RUL segmentectomy. We analyzed the artery composition of the posterior segment to explore the associated pulmonary anatomical features of recurrent artery crossing intersegmental planes.

## Methods

### Patient preparation and reconstruction of 3d-CTBA

Between September 2020 and September 2022, 600 patients with ground-glass opacity were included in the study. These patients underwent routine chest-enhanced CT examinations preoperatively at Hebei General Hospital. The scanning equipment used was the Siemens 64-slice dual-source CT. The whole lung field was scanned with a collimator thickness of 0.6 mm, the reconstructed layer of 1.25 mm, and the interlayer space of 1 mm. A total of 35 ml contrast medium (iopromide 370) was administered intravenously at a rate of 5 ml/s, followed immediately by the intravenous administration of 20 ml of normal saline. By setting a scan start time, the CT value in the pulmonary vein was higher than that in the pulmonary artery and showed a density difference in images. The patients were required to hold their breath throughout the CT scan for appropriate bronchial inflation, and precautions were taken to avoid any potential side effects from the contrast agent following the scan. The volume data from arterial and venous phases were imported into the reconstruction software (InferOperate Thorax Planning), which converted the data into 3D virtual models of the lungs including segments, subsegments, lesions, bronchi, and vessels. Error identification and defects in distal bronchial imaging were manually modified. All procedures involving human participants in this study were in accordance with the Declaration of Helsinki (revised in 2013). This retrospective study was approved by the Research Ethics Committee at Hebei General Hospital (no. 2022119). The need for patient consent was waived because of the retrospective nature of the study.

### Definition of the pulmonary bronchus

**Figure 1:**
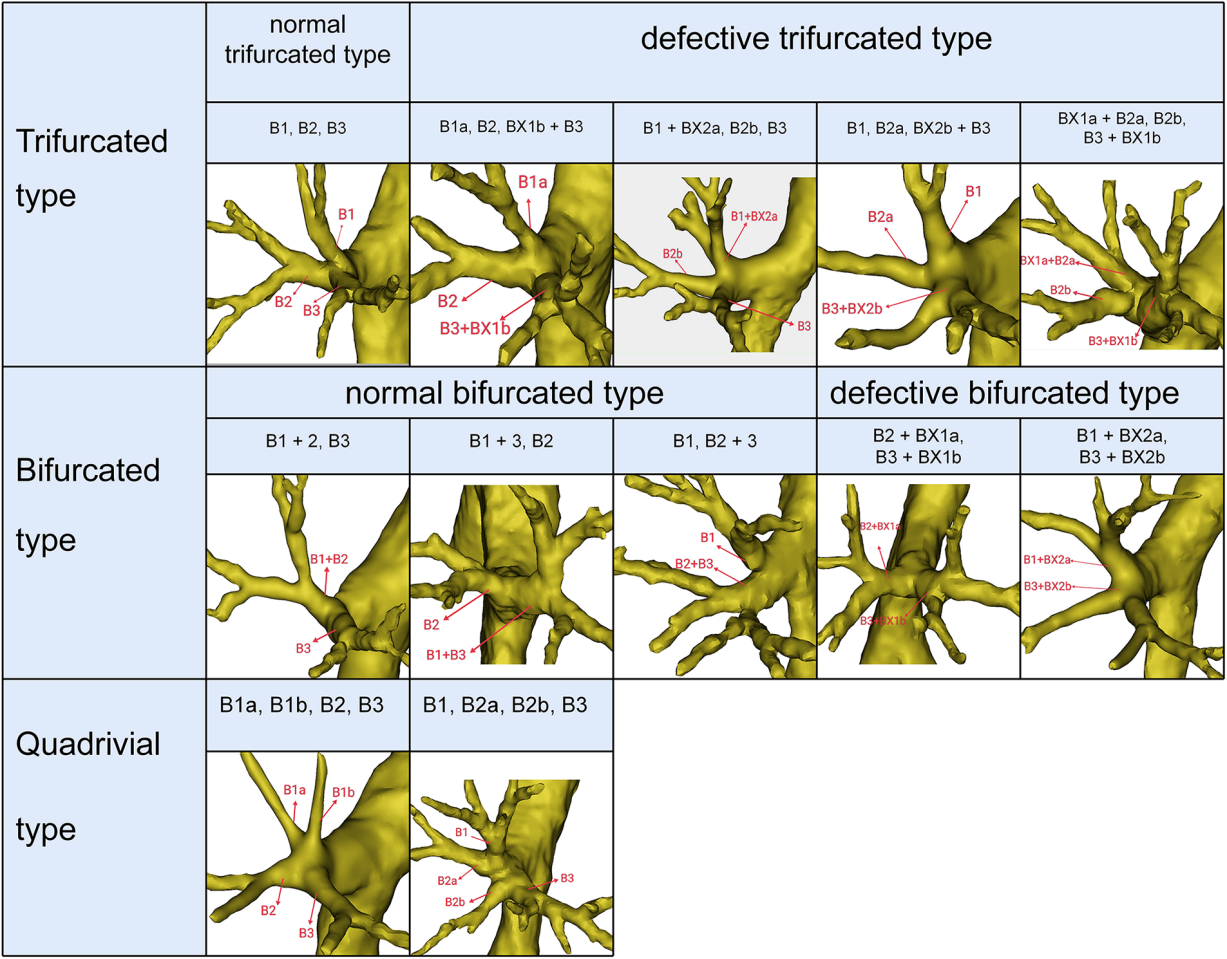
Trifurcated type, bifurcated type, and quadrivial type of RUL bronchus.

Segmental and subsegmental bronchi were named according to the classification proposed by Nagashima (6): B1 is the apical segmental bronchus that divides into apical (B1a) and anterior rami (B1b); B2 is the posterior segmental bronchus that is further divided into posterior (B2a) and horizontal rami (B2b); B3 represents the anterior bronchus that divides into lateral (B3a) and medial rami (B3b). Moreover, the most striking variation in the RUL is the defective bronchus. When S1b was supplied by the defective B1 branching from B3, the defective B1 branch was nominated as BX1b (Figure 1). When S1a was supplied by the defective B1 branching from B2, the defective B1 branch was nominated as BX1a (Figure 1). When S2b was supplied by the defective B2 branching from B3, the defective B2 branch was nominated as BX2b (Figure 1). When S2a was supplied by the defective B2 branching from B1, the defective B2 branch was nominated as BX2a (Figure 1).

**Figure 2:**
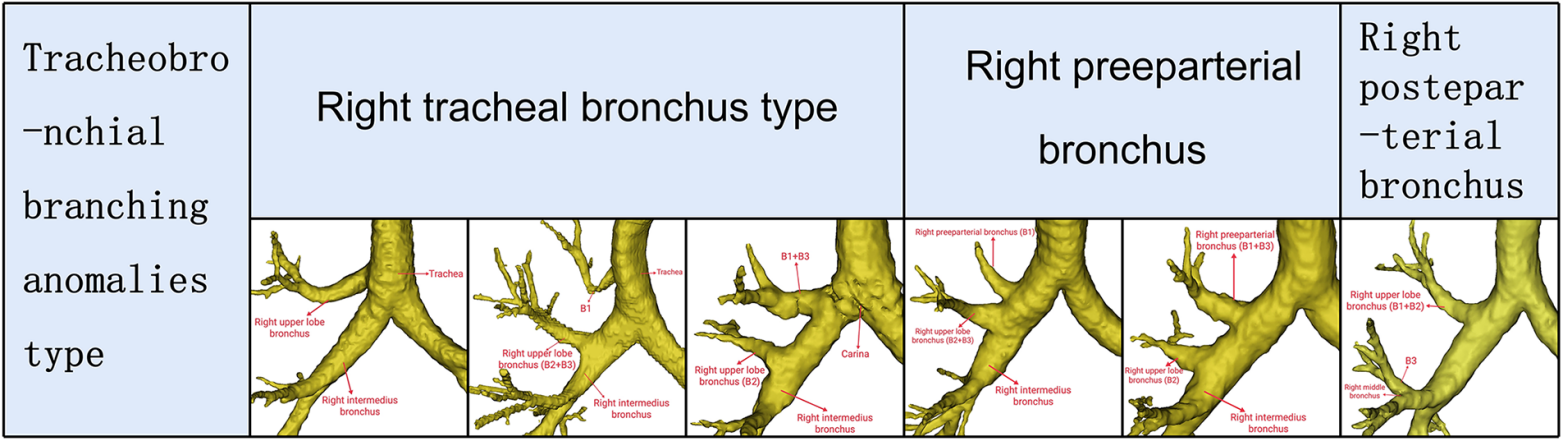
Tracheobronchial branching abnomalies type of RUL bronchus.

In this investigation, the nomenclature used to describe tracheobronchial branching anomalies was Dominique (11). Briefly, the tracheobronchial branching anomalies of the RUL were described as follows: the right tracheal bronchus, the right preeparterial bronchus, and the right posteparterial bronchus (Figure 2). The right tracheal bronchus was described as any bronchus of the RUL that unconventionally arises directly from the lateral wall of the trachea or carina. The right preeparterial bronchus was defined as any bronchus supplying the RUL that originates abnormally from the lateral wall of the right main bronchus above the level of the RUL bronchus. In contrast, any bronchus directed toward the RUL that arises from the right bronchial tree at a level below that of the RUL bronchus was termed the right posteparterial bronchus.

### Definition of the pulmonary artery

**Figure 3:**
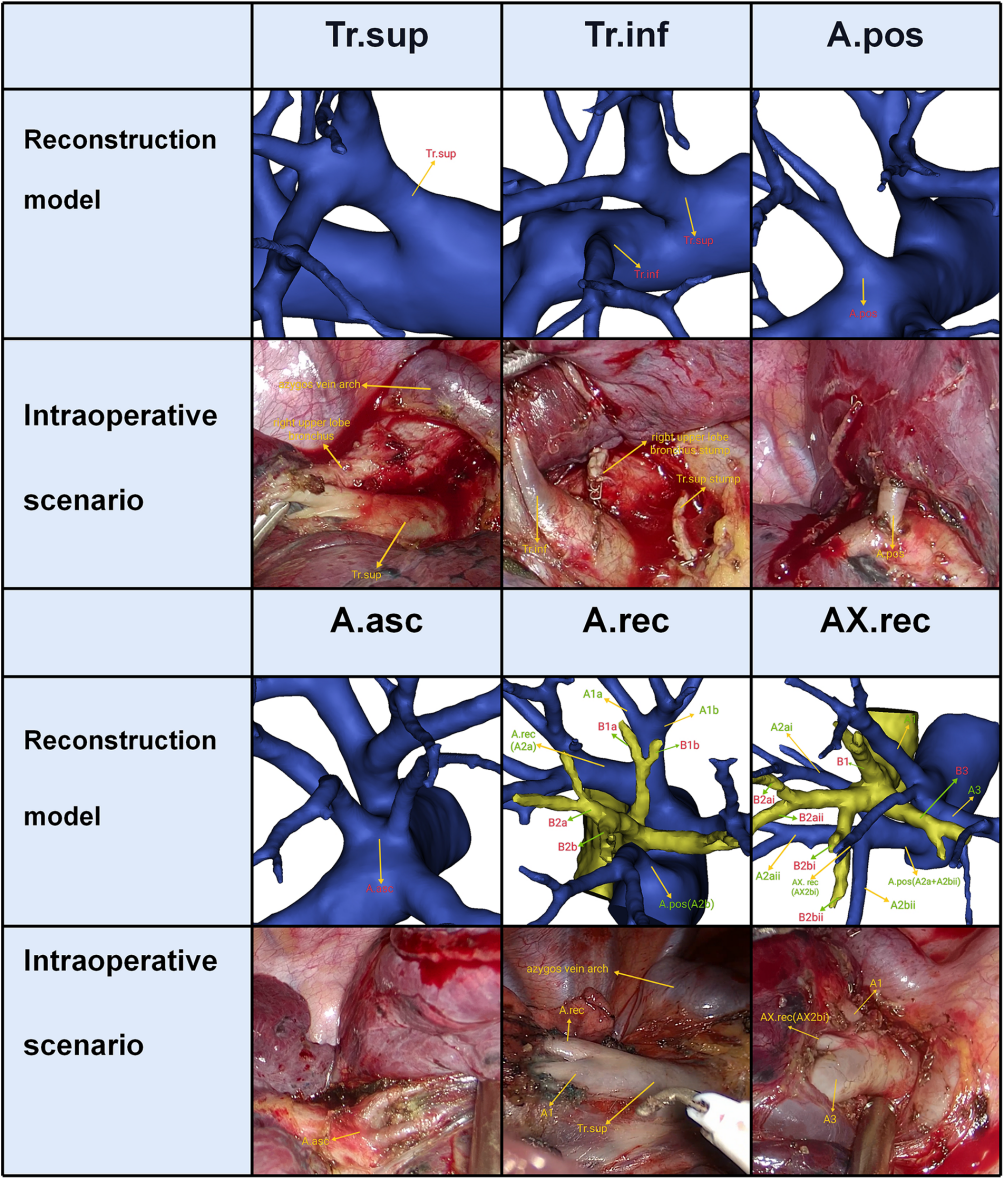
The 3D reconstruction model of the RUL artery.

**Figure 4:**
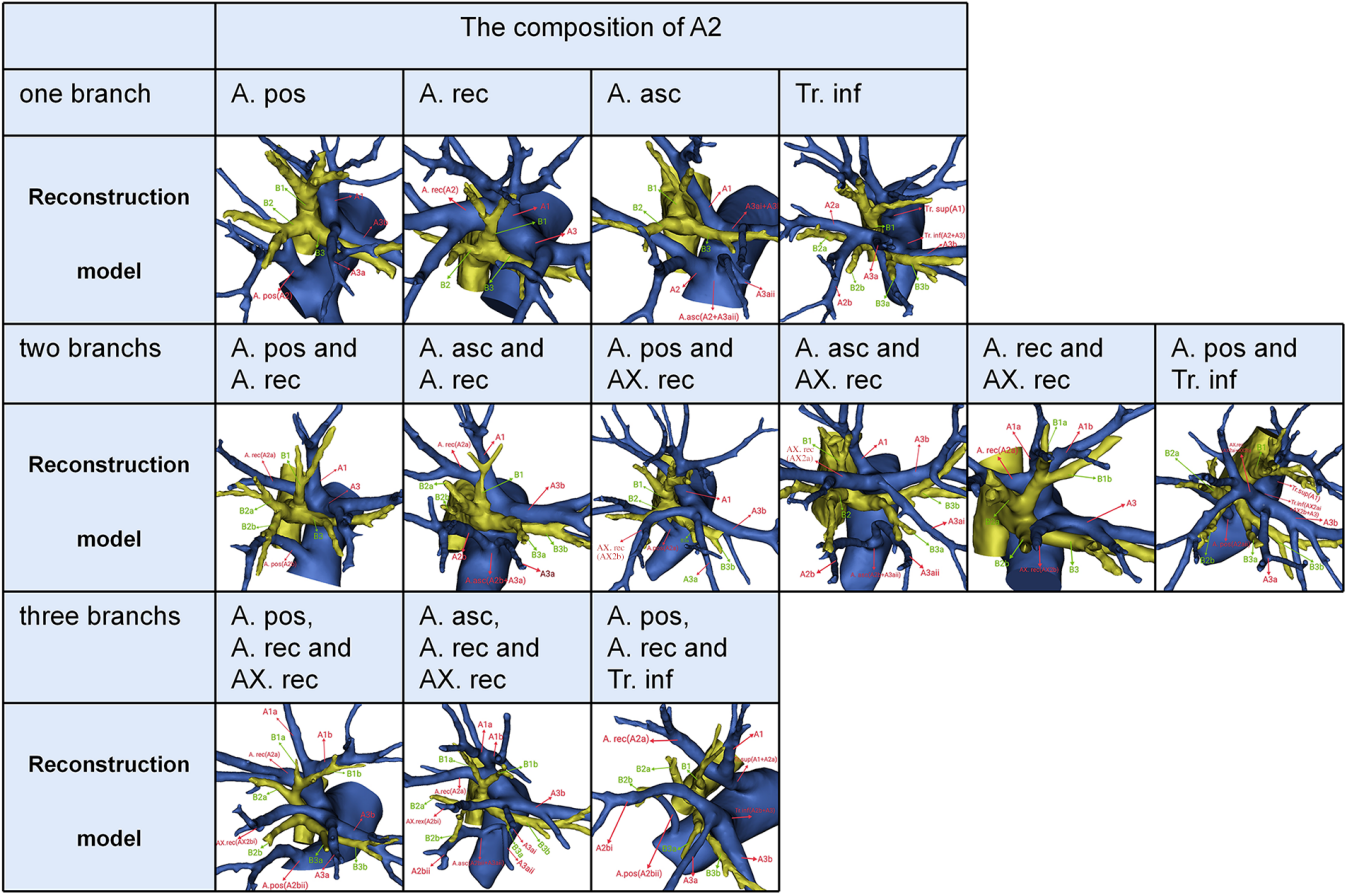
The composition of A2.

Trunk superior (Tr. sup), which lies below the azygos vein arch and flows into RUL at the anterior side of the RUL bronchus, is the first branch of the right main pulmonary artery and is often the chief source of the RUL artery (Figure 3). Trunk inferior (Tr. inf) also originates from the mediastinal portion of the artery and passes anteriorly to B3 (Figure 3). The recurrent artery (A. rec) is a branch of Tr. sup that crosses behind the B1a to supply S2 (Figure 3). Recurrent artery crossing intersegmental planes were defined AX. rec that originates from Tr. sup and crosses between B1 and B3 to supply the S2 (Figure 3). The artery branch originating from the interlobar portion of the right pulmonary artery locates at the posterior side of B3. The posterior ascending artery (A. pos) is named if it supplies only the S2 (Figure 3), and the ascending artery (A. asc) is named if it supplies both S2 and S3 (Figures 3, 4).

### Statistics

All statistical analyses were performed using SPSS 23.0 (SPSS, Chicago, IL, USA). Qualitative data were expressed as the number of cases (percentage). Comparison between groups was assessed by using the Pearson Chi-Square test. A *P*-value less than 0.05 was considered indicative of a significant difference.

## Results

The mean age of 600 patients (336 females and 264 males) was 58 years. Four types could be defined according to the branches of the RUL bronchus (Table 1, Figures 1, 2): trifurcated type (315/600, 52.5%), bifurcated type (239/600, 39.8%), quadrivial type (33/600, 5.5%), and tracheobronchial branching abnormalities type (13/600, 2.2%). The trifurcated type included the normal trifurcated type (275/600, 45.8%), which is regarded as the most common type of the RUL bronchus, and the defective trifurcated type, which component contained the defective bronchus (Figure 1). The defective trifurcated type could be further separated into the following four types: B1a, B2, BX1b + B3 type, B1 + BX2a, B2b, B3 type, B1, B2a, BX2b + B3 type, and BX1a + B2a, B2b, B3 + BX1b type. The bifurcated type was also divided into the normal bifurcated type and the defective bifurcated type (Figure 1). The normal bifurcated type was further subclassified into the following three subtypes: subtype I (B1 + 2, B3), subtype II (B1 + 3, B2), and subtype III (B1, B2 + 3). The defective bifurcated type, which component also included the defective bronchus, could be further subclassified into two types: the defective B1 type (B2 + BX1a, B3 + BX1b) and the defective B2 type (B1 + BX2a, B3 + BX2b). The quadrivial type contained B1a, B1b, B2, B3 type and B1, B2a, B2b, B3 type.

**Table 1:** Bronchus type.

	Our study (*n* = 600)		Nagashima (*n* = 263)	
	No.	%	No.	%
Trifurcated type	315	52.5	116	44.1
Normal trifurcated type (B1, B2, B3)	275	45.8	116	44.1
Defective trifurcated type	40	6.7	NR	–
B1a, B2, BX1b + B3	22	3.7	NR	–
B1 + BX2a, B2b, B3	11	1.8	NR	–
B1, B2a, BX2b + B3	3	0.5	NR	–
BX1a + B2a, B2b, B3 + BX1b	4	0.7	NR	–
Bifurcated type	239	39.8	96	36.5
Normal bifurcated type	146	24.3	77	29.3
B1 + 2, B3	62	10.3	38	14.4
B1 + 3, B2	41	6.8	23	8.8
B2 + 3, B1	43	7.2	16	6.1
Defective bifurcated type	93	15.5	19	7.2
B2 + BX1a, B3 + BX1b	75	12.5	13	4.9
B1 + BX2a, B3 + BX2b	18	3.0	6	2.3
Quadrivial type	33	5.5	2	0.8
B1a, B1b, B2, B3	4	0.7	2	0.8
B1, B2a, B2b, B3	29	4.8	NR	–
Tracheobronchial branching anomalies type	13	2.2	NR	–
Right tracheal bronchus	4	0.7	NR	–
Right preeparterial bronchus	7	1.2	NR	–
Right posteparterial bronchus	2	0.3	NR	–
N/A	–	–	49	18.6

N/A, not available; NR, the type was not referred.

The tracheobronchial branching anomalies type had three forms as following: the right tracheal bronchus type, the right preeparterial bronchus type, and the right posteparterial bronchus type. In the right tracheal bronchus type, the RUL bronchus originating from the lateral wall of the trachea was found in 2 cases (Figure 2); the B1 segmental bronchus originating from the lateral wall of the trachea and B2 and B3 segmental bronchi originating from the RUL bronchus was discovered in 1 case (Figure 2); the B1 and B3 segmental bronchi originating from the lateral wall of the carina and B2 segmental bronchus originating from the RUL bronchus was also observed in 1 case (Figure 2). In the right preeparterial bronchus type, B1 segmental bronchus arising from the right preeparterial bronchus and B2 and B3 segmental bronchi arising from the RUL bronchus were found in 4 cases (Figure 2); B1 and B3 segmental bronchi arising from the right preeparterial bronchus and B2 arising from the RUL bronchus were discovered in 3 cases (Figure 2). Moreover, B1 and B2 segmental bronchi arising from the RUL bronchus and B3 segmental bronchus arising from the right middle bronchus were seen in 2 cases in the right posteparterial bronchus type (Figure 2).

The composition of A2 was complex. According to the branches of A2, three categories could be defined (Table 2, Figure 4). First, A2 was only supplied by one branch of the artery: A. pos, A. rec, A. asc, and Tr. inf. Second, A2 was supplied by two branches of the artery: A. pos and A. rec; A. asc and A. rec; A. pos and AX. rec; A. asc and AX. rec; A. rec and AX. rec; A. pos and Tr. inf. Third, A2 was supplied by three branches of the artery: A. pos, A. rec, and AX. rec; A. asc, A. rec, and AX. rec; A. pos, A. rec, and Tr. inf. As detailed in Table 2, the incidence of AX. rec was 12.7% (70/600). Furthermore, we summarized the distribution of AX. rec in bronchus type (Table 3).

**Table 2:** The composition of A2.

	Our study (*n* = 600)	
	No.	%
One branch	193	32.1
A. pos	110	18.3
A. rec	57	9.5
A. asc	24	4.0
Tr. inf	2	0.3
Two branchs	385	64.2
A. pos and A. rec	240	40.0
A. asc and A. rec	91	15.2
A. pos and AX. rec	37	6.2
A. asc and AX. rec	9	1.5
A. rec and AX. rec	3	0.5
A. pos and Tr. inf	5	0.8
Three branchs	22	3.7
A. pos, A. rec and AX. rec	17	2.8
A. asc, A. rec and AX. rec	4	0.7
A. pos, A. rec and Tr. inf	1	0.2

**Table 3:** Distribution of AX.rec in bronchus type.

	With AX.rec	Without AX.rec	Total
**Bronchus type**			
B1, B2, B3	30	245	275
B1a, B2, BX1b + B3	5	17	22
B1 + BX2a, B2b, B3	1	10	11
B1, B2a, BX2b + B3	1	2	3
BX1a + B2a, B2b, B3 + BX1b	0	4	4
B1 + 2, B3	5	57	62
B1 + 3, B2	0	41	41
B2 + 3, B1	11	32	43
B2 + BX1a, B3 + BX1b	1	74	75
B1 + BX2a, B3 + BX2b	6	12	18
B1a, B1b, B2, B3	1	3	4
B1, B2a, B2b, B3	8	21	29
Right tracheal bronchus	0	4	4
Right preeparterial bronchus	1	6	7
Right posteparterial bronchus	0	2	2
Total	70	530	600

**Figure 5:**
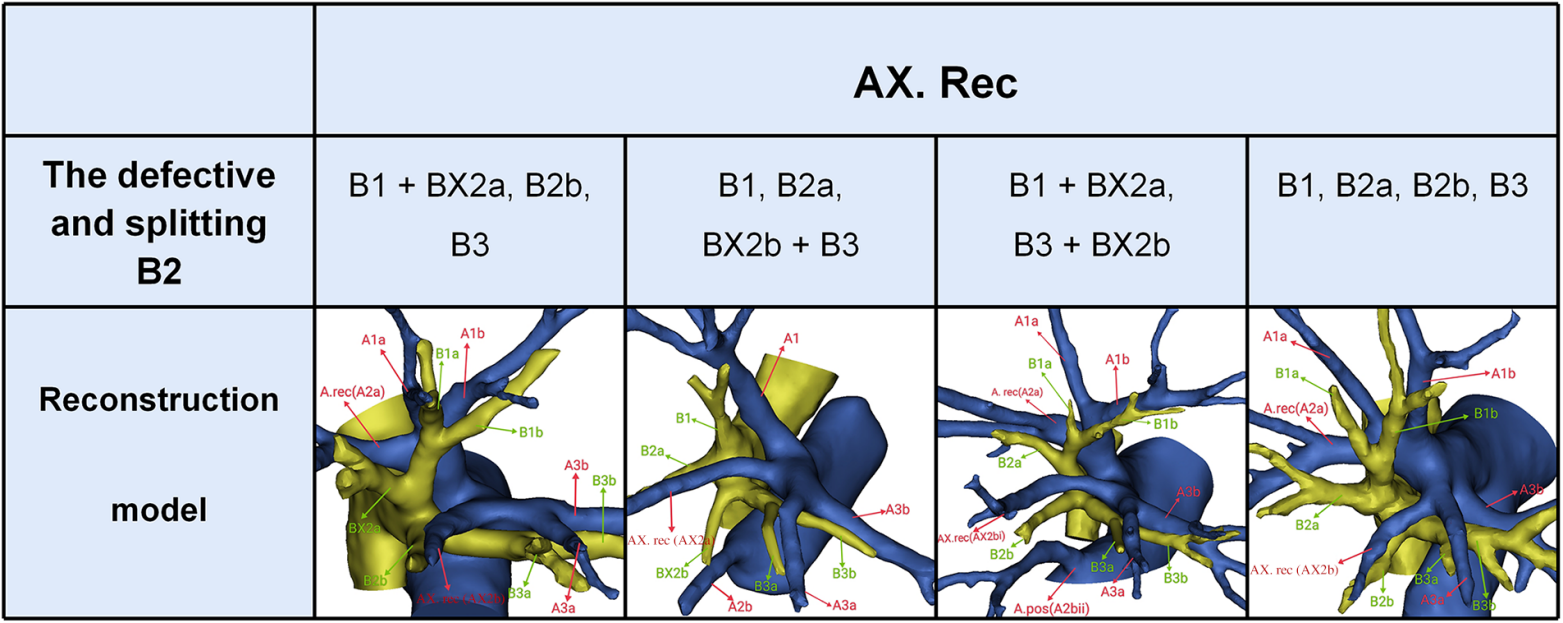
The distribution of AX. rec in the defective and splitting B2 type.

In the following types, B1 + BX2a, B2b, B3 (11/600, 1.8%), B1, B2a, BX2b + B3 (3/600, 0.5%), and B1 + BX2a, B3 + BX2b (18/600, 3%), B2 comprised the defective bronchus branch, including BX2a and B2b, B2a and BX2b, and BX2a and BX2b, respectively (Figure 1). In B1, B2a, B2b, B3 type (29/600, 4.8%), B2 was split into two segments at the root; thus, B2a and B2b originated from the RUL bronchus (Figure 1). Therefore, the incidence of B2 defective or splitting was 10.2% (61/600). As shown in Figure 5 and Table 4, the incidence of AX. rec with and without the defective and splitting B2 was 26.2% (16/61) and 10.0% (54/539), respectively (*p* < 0.005). This indicates that the incidence of AX. rec was increased in patients with defective and splitting B2.

**Table 4:** Distribution of AX.rec in the defective and splitting B2 type.

	With AX.rec	Without AX.rec	Total	*p* value
With the defective and splitting B2	16	45	61	*p* < 0.005
Without the defective and splitting B2	54	485	539	
Total	70	530	600	

## Discussion

With the prevalence of thoracic thin-section CT, the detection rate of GGO increases, and some research results have indicated that the prognosis of segmentectomy is not worse than that of lobectomy in patients with early lung cancer (1–5). The JCOG0804 study, which assessed the effectiveness and safety of sublobar resection for peripheral lung cancer with consolidation tumor ratio ≤ 0.25 and maximum tumor diameter ≤ 2.0 cm, has clarified that sublobar resection was the preferred surgical procedure as long as the adequate surgical margin was available (1). In addition, sublobar resection provides valuable surgical opportunities for patients who could not undergo standard lobectomy because of the limited cardiopulmonary reserve. However, the anatomical variation of the bronchus is complex. In the past, the anatomical variation of the bronchus was explored through gross anatomical specimens, tracheoscopy, and 2D CT images. Nevertheless, a more accurate and detailed anatomical analysis could not be implemented due to the lack of 3D picture images. Fortunately, advances in the volume-rendering reconstruction technique have enabled the reconstruction of 3D images. 3D-CTBA, a useful tool for thoracic surgeons to identify pulmonary anatomy more intuitively compared with conventional CT images, assists in a comprehensive understanding of the pulmonary anatomy of each patient before and during surgical procedures. In the present study, we systematically analyzed the RUL bronchus and the artery composition of S2 by 3D-CTAB imaging. Furthermore, we explored the associated pulmonary anatomical features of AX. rec.

In this study, the trifurcated type was seen in 52.5% of cases (Table 1), which was considerably higher than the frequency reported by Nagashima (6) (44.1%). We found that the trifurcated type had two branching types (Table 1): the normal trifurcated type (45.8%), which incidence was higher than that of Nagashima (44.1%), and the defective trifurcated type (6.7%), which has not been reported in the literature. Then, the defective trifurcated type was subdivided into four subtypes. The classification of the bifurcated bronchus type was the same as that of Nagashima (Table 1). Moreover, we found that the quadrivial type had two branching types (Table 1): B1a, B1b, B2, B3 (0.7%), which incidence was similar to that of Nagashima (0.8%), and B1, B2a, B2b, B3, which has not been reported in the literature. A detailed classification and occurrence probability of tracheobronchial branching anomalies type have been rarely involved in previous studies. In this paper, the tracheobronchial branching anomalies of the RUL are classified and summarized in detail (Table 1).

In anatomical segmentectomy, it is extremely significant to master these new variation types of the segmental and subsegmental bronchi. For B1a, B2, B3 + BX1b type, we should separate BX1b from B3 when we perform an accurate S1 segmentectomy (Figure 1). For B1 + BX2a, B2b, B3 type, B1 needs to be separated to the subsegment level to retain the defective B2 branch supplied to S2 when S1 segmentectomy is planned (Figure 1). When S3 segmentectomy is performed for B1, B2a, BX2b + B3 type (Figure 1), it is necessary to dissect B3 to the subsegment level because BX2b originates from B3. When S2a segmentectomy is performed for BX1a + B2a, B2b, B3 + BX1b type (Figure 1), a mistaken cut at the trunk of BX1a + B2a will result in an enlarged intersegmental plane. B2a and B2b respectively originate from the RUL bronchus in B1, B2a, B2b, B3 type. Thus, it is extremely easy to mistaken B2a or B2b for B2 during S2 segmentectomy, which will narrow the intersegmental plane (Figure 1).

Although only a few surgical cases of lung cancer involving tracheobronchial branching abnormalities have been reported (12–14), it is usually more challenging for thoracic surgeons and anesthetists to perform a video-assisted thoracoscopic surgery (VATS) lobectomy. For example, when the posteparterial bronchus is B3 (11, 13, 14), there is often an incomplete horizontal fissure, with A3 descending to the artery of the right middle lobe (Figure 2). When the preeparterial bronchus is B1 (11, 12), there is often a pulmonary vein variant, which lays behind the trunk of the pulmonary artery and flows into the left atrium (Figure 2). In this case, to successfully complete the VATS lobectomy, surgeons should pay attention to the preoperative image understanding and intraoperative scenario verification. The anatomical anomaly of the bronchus that is most relevant to anesthesiology practice is the tracheal bronchus (15). Moreover, the anesthesiologist easily fails when they try to place double-lumen endotracheal intubation due to the presence of tracheal bronchus (Figure 2).

**Figure 6:**
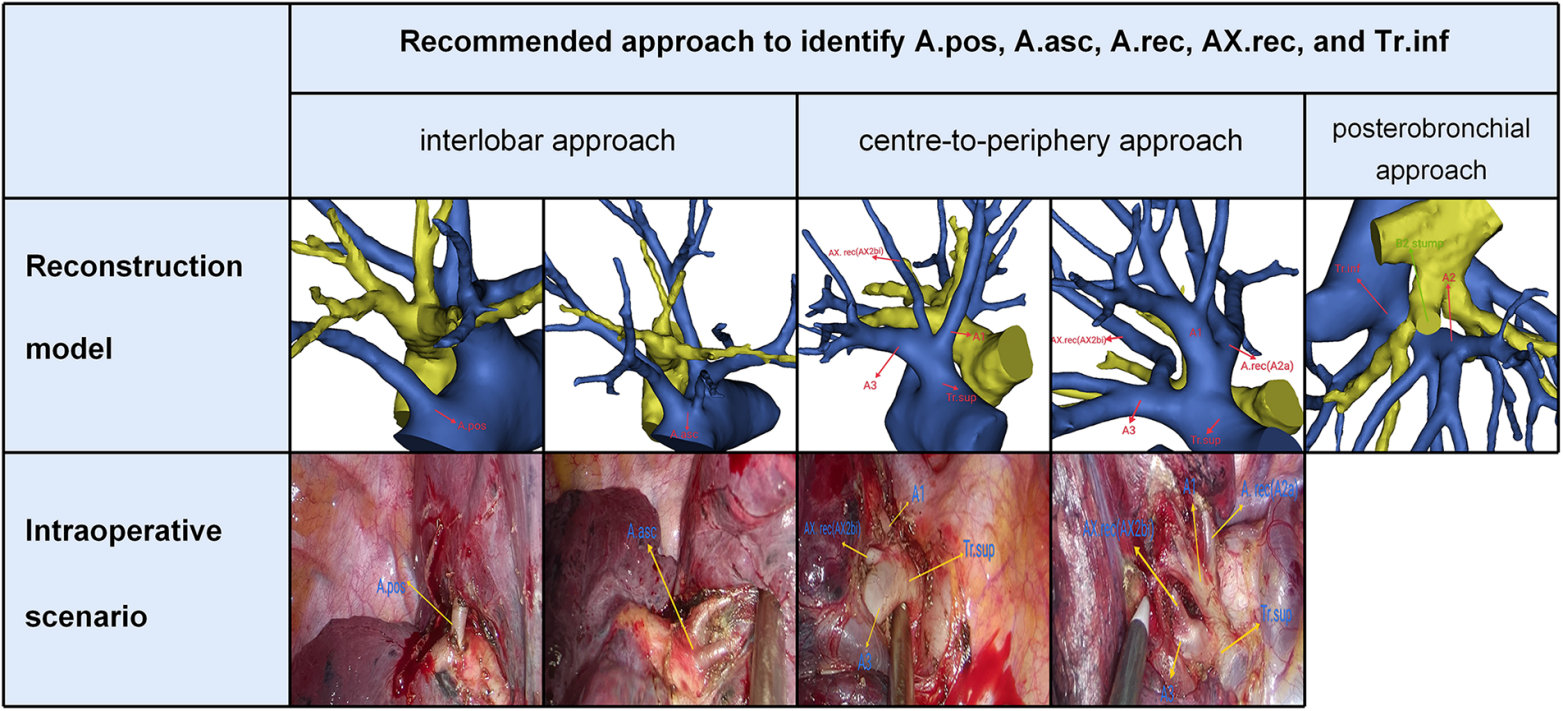
Three basic approaches to identify A.pos, A.asc, A.rec, AX.rec, and Tr.inf.

In the present study, five components of A2 composition were summarized as follows (Figure 4, Table 2): A. pos, A. asc, A. rec, AX. rec, and Tr. inf. Therefore, three basic approaches to identifying these branches were first reported (Figure 6). The interlobar approach was adopted to distinguish arteries that originate from the interlobar portion of the right pulmonary artery. Center-to-periphery approach was used to identify artery branches that derive from the Tr. sup. A posterobronchial approach was applied to dissect the artery branch running deep within the lung parenchyma. Therefore, A. pos and A. asc can usually be identified by dissecting interlobar fissures (interlobar approach). Tr. sup can be dissected in a center-to-periphery direction to distinguish A. rec and AX. rec (center-to-periphery approach). Tr. inf running deep within the lung parenchyma and supplying S2 was identified after resection of the targeted segmental bronchus (posterobronchial approach). The comprehensive understanding of these basic approaches has clinical significance for S2 segmentectomy. Moreover, the branching pattern of A2 has significant clinical significance for accurate S2 segmentectomy (Figure 4, Table 2). If A2 originates from A. pos (Figure 4), A. pos is dissected by adopting the interlobar approach. If A2 has two branches of A. pos and A. rec (Figure 4), we need to apply the interlobar approach and center-to-periphery approach to discriminate A. pos and A. rec. If A2 has three branches of A. pos, A. rec, and Tr. inf, the interlobar approach, center-to-periphery approach, and posterobronchial approach were respectively adopted to dissect A. pos, A. rec, and Tr. inf.

Another interesting finding of this study concerned AX. rec (Figure 5, Tables 3, 4). AX. rec is not rare in clinical practice; however, if it occurs, it causes great difficulties in performing pulmonary segmental resection in patients. However, AX. rec has not been reported in previous studies about bronchovascular variations of the RUL. Coincidently, the incidence of AX. rec with and without the defective and splitting B2 was 26.2% (16/61) and 10.0% (54/539), respectively (*p* < 0.005). This indicates that the incidence of AX. rec was increased in patients with defective and splitting B2. This may be interpreted by the paralleling relationship between pulmonary segmental arteries and pulmonary segmental bronchi. Similarly, this AX. rec might result in a slight shift for the intersegmental plane between S2a and S2b. Thus, when the AX. rec is discovered intraoperatively, it is crucial to keep an eye on the existence of B2 defective and splitting. For S1 segmentectomy, it is necessary to dissect Tr. sup from the central to the peripheral direction to disconnect A1 and protect AX. rec (Figure 5). Additionally, we found that AX. rec shared a common trunk with A3. When S3 segmentectomy is performed, a mistaken cut at the trunk of AX. rec and A3 will result in an enlarged intersegmental plane. Therefore, an understanding of the branches and direction of AX. rec before surgery allows the surgeon to carefully peel off A3 intraoperatively, avoiding injury to AX. rec (Figure 5). In sum, we defined the coexistence of AX. rec and B2 defective or AX. rec and B2 splitting as “Hebei's two combinations.”

## Conclusions

This is the first report to explore the associated pulmonary anatomical features of AX. rec. We found that the incidence of AX. rec was increased in patients with defective and splitting B2. This knowledge will assist in the preoperative planning of RUL segmentectomy.

## Data Availability

The raw data supporting the conclusions of this article will be made available by the authors, without undue reservation.
